# Supplementary material for: Episodic back-arc spreading centre jumps controlled by transform fault to overriding plate strength ratio
Source: Nat Commun. 2022 Jan 31;13:582. doi: 10.1038/s41467-022-28228-5 (PMC8803885; doi:10.1038/s41467-022-28228-5)
Supplement: Supplementary file 1 — Supplementary Information [file 41467_2022_28228_MOESM1_ESM.pdf]

Episodic back-arc spreading centre jumps controlled by transform fault to overriding plate strength ratio

SUPPLEMENTARY INFORMATION

Nicholas Schliffke[1] (nico.schliffke@web.de), Jeroen van Hunen[1], Mark B. Allen[1], Valentina Magni[2], Frédéric Gueydan[3]

[1]Department of Earth Sciences, Durham University, DH1 3LE, Durham, United Kingdom;

[2]The Centre for Earth Evolution and Dynamics (CEED), University of Oslo, SemSaelandsvei 24, PO Box 1048, Blindern, NO-0316 Oslo, Norway

[3]Géosciences Montpellier, Université Montpellier, place E. Bataillon 34095 Montpellier cedex 5, France

SUPPLEMENTARY FIGURES

Here, we present additional information to support the models presented in the main article.

Initial model evolution of the reference model

Supplementary Fig. 1 shows the initial stages of the reference model with subduction between the prescribed transform faults. The slab sinks under its own buoyancy and the subduction systems rolls back (see Figure Supplementary Fig. 2). Initial subduction and rollback can occur without much friction between the transform faults in all models due to decoupling of subducting and overriding plate from neighbouring plates. Once rollback reaches the end of the pre-imposed transform faults, further rollback of the subducting slab is only feasible by tearing of lithosphere between subducting and neighbouring plates at STEP faults. At this point, the first back-arc basin is formed: high tear resistance at the STEP faults reduce lateral rollback at the plate interface, and the resulting trench curvature localizes stresses and ruptures the upper plate.

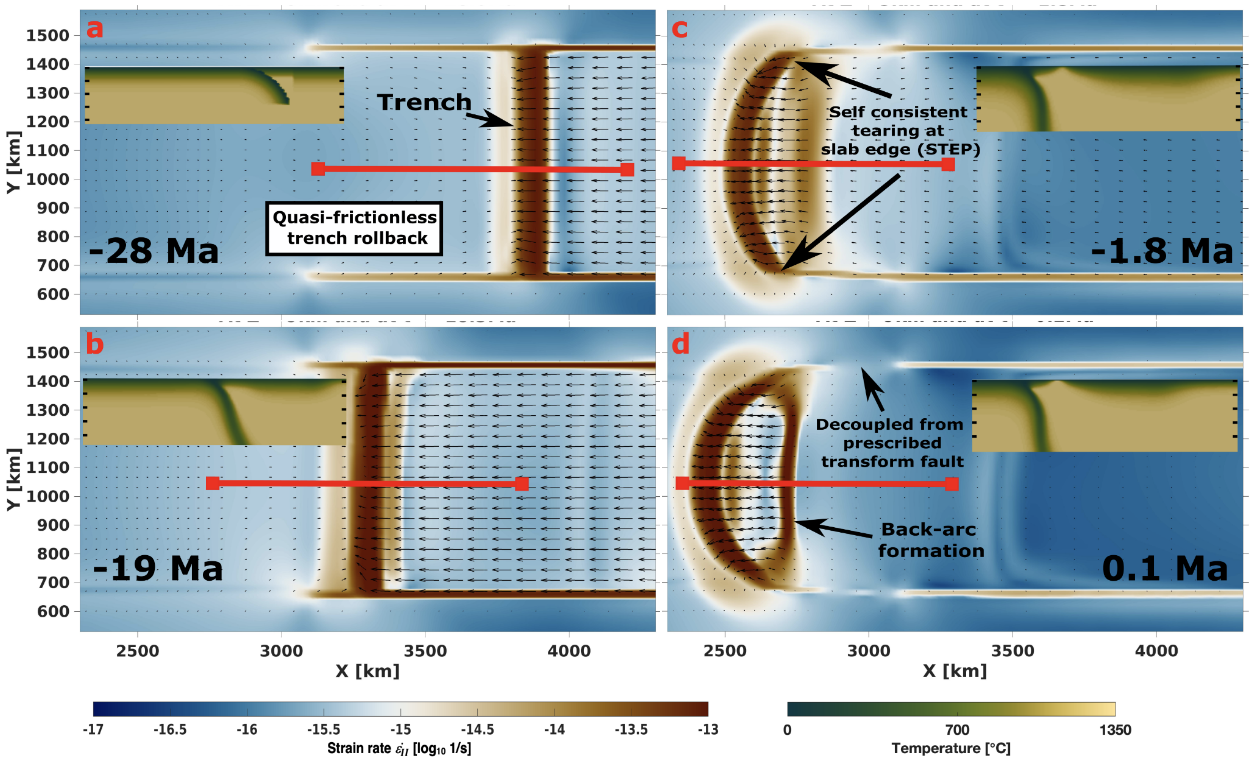

Supplementary Figure 1. Initial evolution. *Initial model evolution until opening of first back-arc basin. Colours refer to the 2nd invariant of the strain rate at the surface in plan view, while insets show vertical cross sections of the temperature field below the red lines.* ***a+b)*** *Within prescribed transform faults, the trench retreats without formation of a back-arc basin due to decoupling from neighbouring plates.* ***c+d)*** *Once beyond the pre-imposed transform faults, the subducting slab tears along neighbouring continents self-consistently by localising stresses at slab edges (STEPs) and back-arc spreading commences. The moment the back-arc spreading centre develops is defined as time t=0 which is comparable to opening of back-arc basins in the models of* Magni et al., (2014). *The model is thus suitable to study the dynamics of following back-arc spreading centre jumps from this point.*

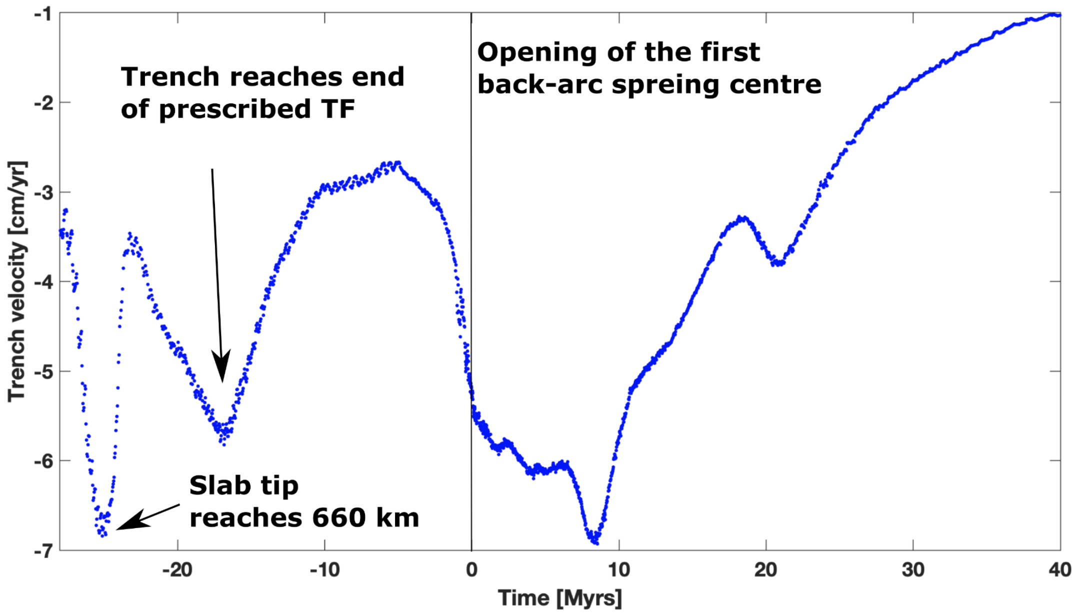

Supplementary Figure 2*. *Entire model evolution. *Trench rollback for the entire reference model with t=0 scaled to opening of the initial opening of a back-arc spreading centre. Most rapid rollback rates occur during the initial sinking phase of the slab until it reaches the 660 km and shortly after opening of the first back-arc spreading centre. See Figure 3 for details after t=0.*

The role of the toroidal flow for localisation of stress during the back-arc spreading jumps

Once a back-arc spreading centre jump is about to take place, the location of the new spreading centre will be, in part, determined, by the local stress state of the overriding plate. The new back-arc spreading localises where previously the toroidal flow begins to drag overriding lithosphere (see streamlines at the base of the lithosphere) and hence induces the highest strain.

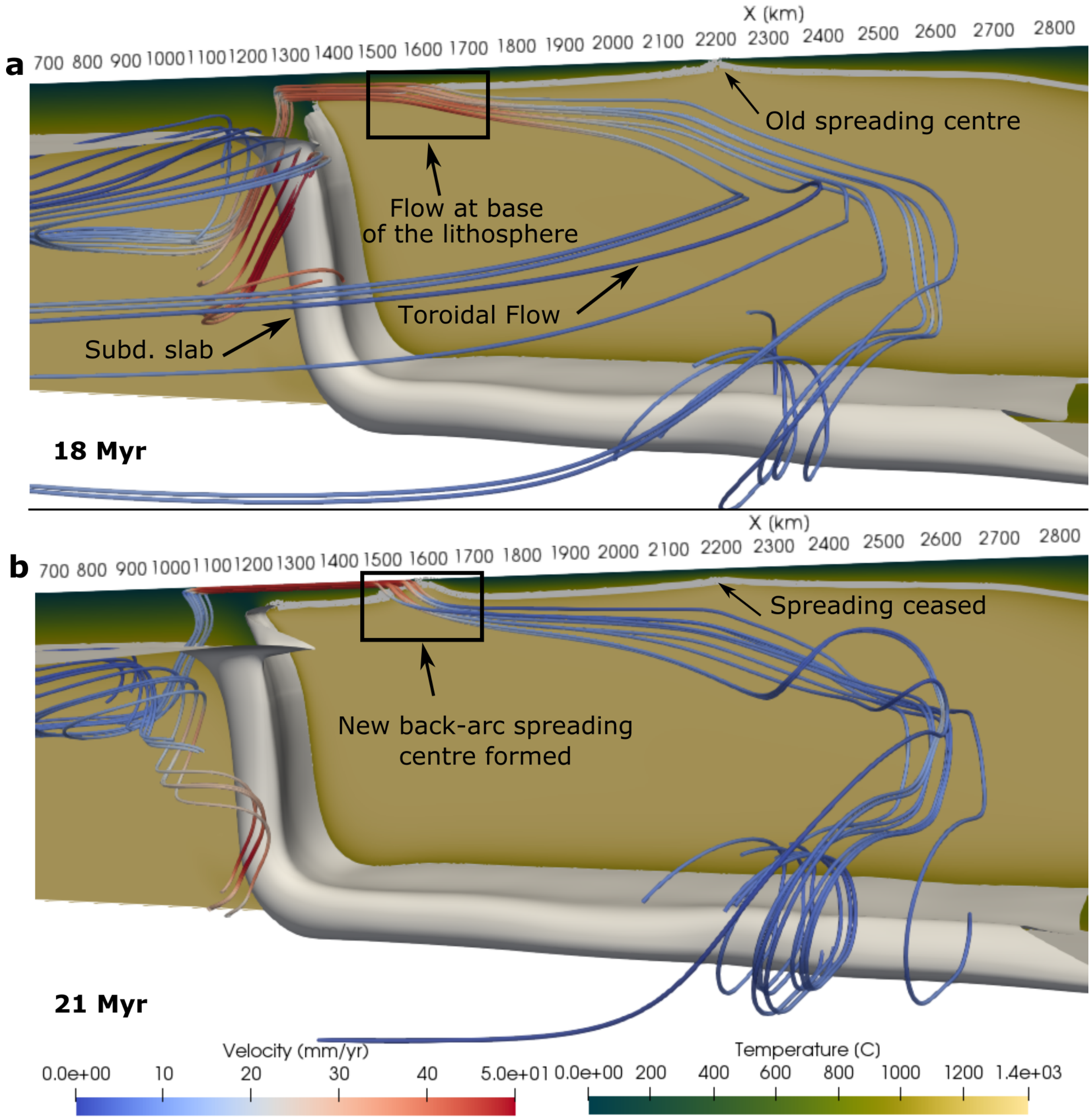

Supplementary Figure 3. Mantle Flow. *The toroidal flow determining the location of the new spreading centre in the reference model (800km wide plate) at model time t=18 Myr (a) and 21 Myr (b). Grey shows the subducting slab, the vertical cross section shows a temperature profile through the centre of the domain. The streamlines (i.e. integrated velocities) are colour-coded by the magnitude of the current velocities.*

The evolution of the models without spreading centre jumps

Below we present the three models to support our conclusion that the propagation of STEP faults and a limited length of transform faults are crucial for back-arc spreading jumps:

1) The model with a 400 km wide slab is unable to overcome tear resistance at STEP faults due to insufficient stress localisation at the slab edges to weaken the lithosphere. This prevents rollback and subduction completely (Supplementary Fig. 4).

2) In contrast, the 1500 km wide slab does form a back-arc spreading centre as well as STEP and transform faults. However, the slab retreats continuously until it reaches the model edge without a spreading jump (Supplementary Fig. 5).

3) No spreading jumps develop in models with prescribed weak zones (i.e. transform faults) along the entire neighbouring plates (Supplementary Fig. 6). With prescribed weaknesses, both the slab and overriding plate can retreat along neighbouring plate effortlessly and strain rate localisation is spread along the entire plate margin. In this setting, stress localisation is not required to weaken the lithosphere for ongoing subduction and rollback. The subduction system retreats thus with the entire overriding plate and no back-arc spreading centre is formed at all.

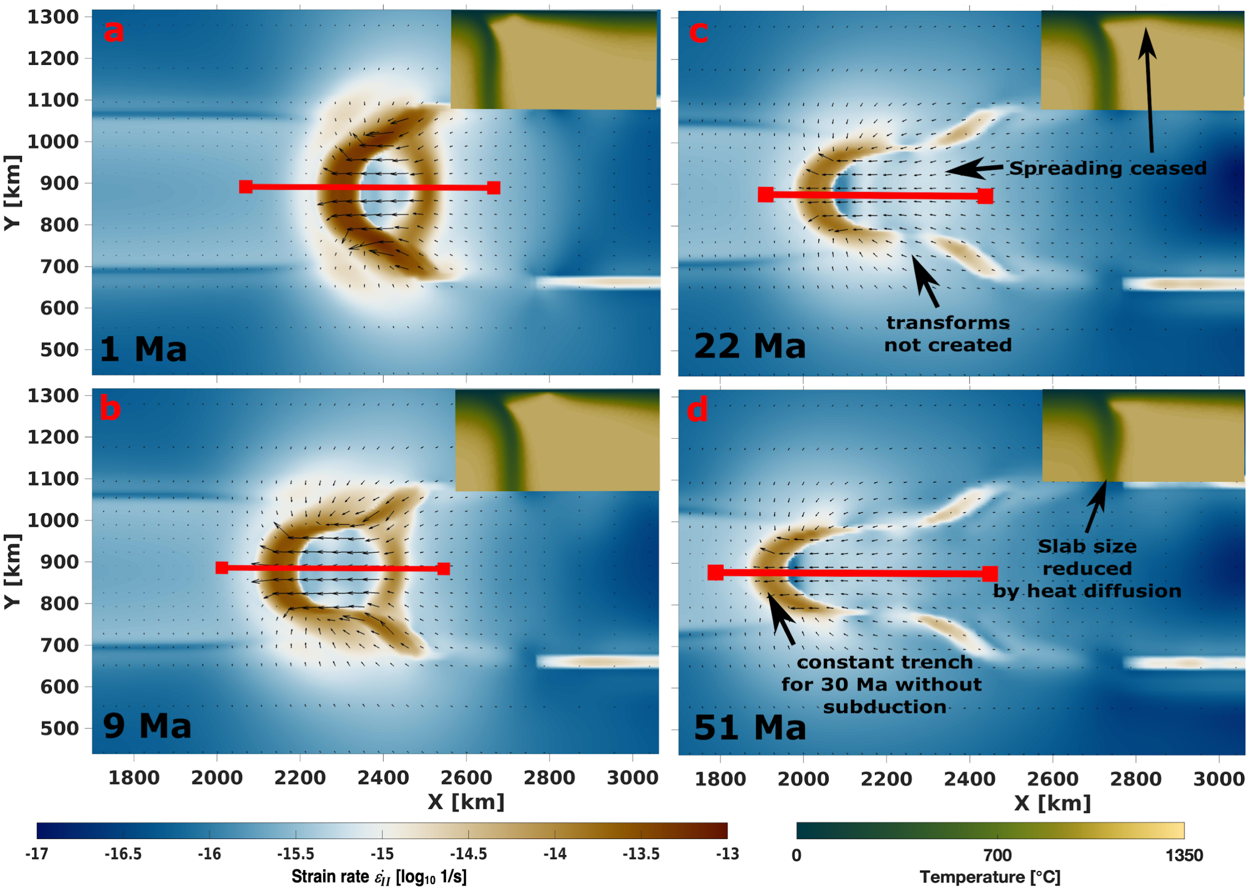

Supplementary Figure 4. Narrow plate model. *Model evolution for a 400 km wide slab.* ***a+b)*** *After formation of the first back-arc spreading centre, the trench remains nearly stationary* ***(c+d)*** *without further subduction. No transform faults connecting the trench and back-arc spreading centre are formed. The back-arc is 'sealed' by cooling and the slab heats up diffusively in the mantle.*

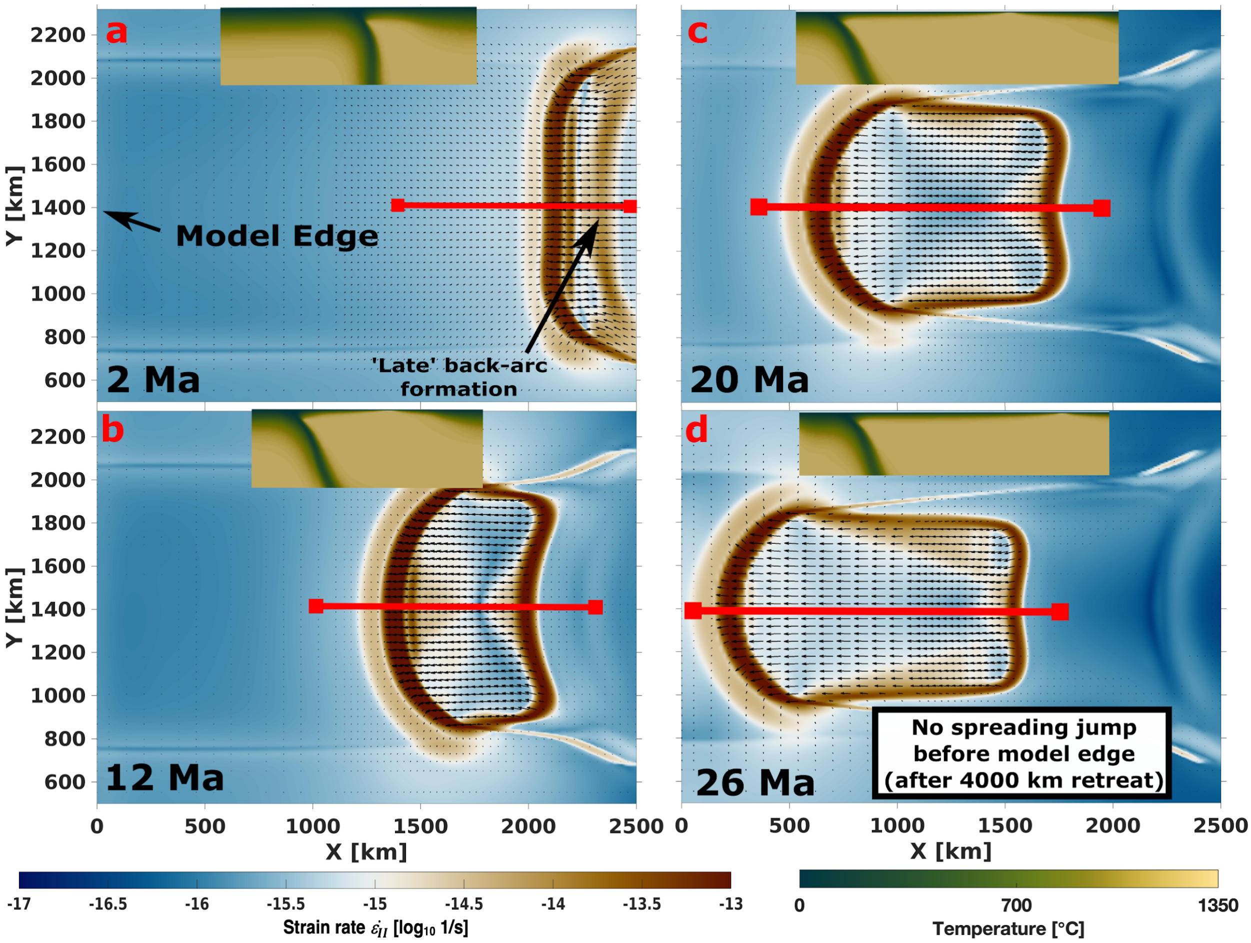

Supplementary Figure 5*. *Wide plate model. *Model evolution for a 1500 km wide slab with no back-arc spreading jump.* ***a)*** *The first back-arc basin is formed late (after 2000 km trench retreat).* ***b-d)*** *Ongoing rollback does not cause a back-arc spreading jump within the model domain.*

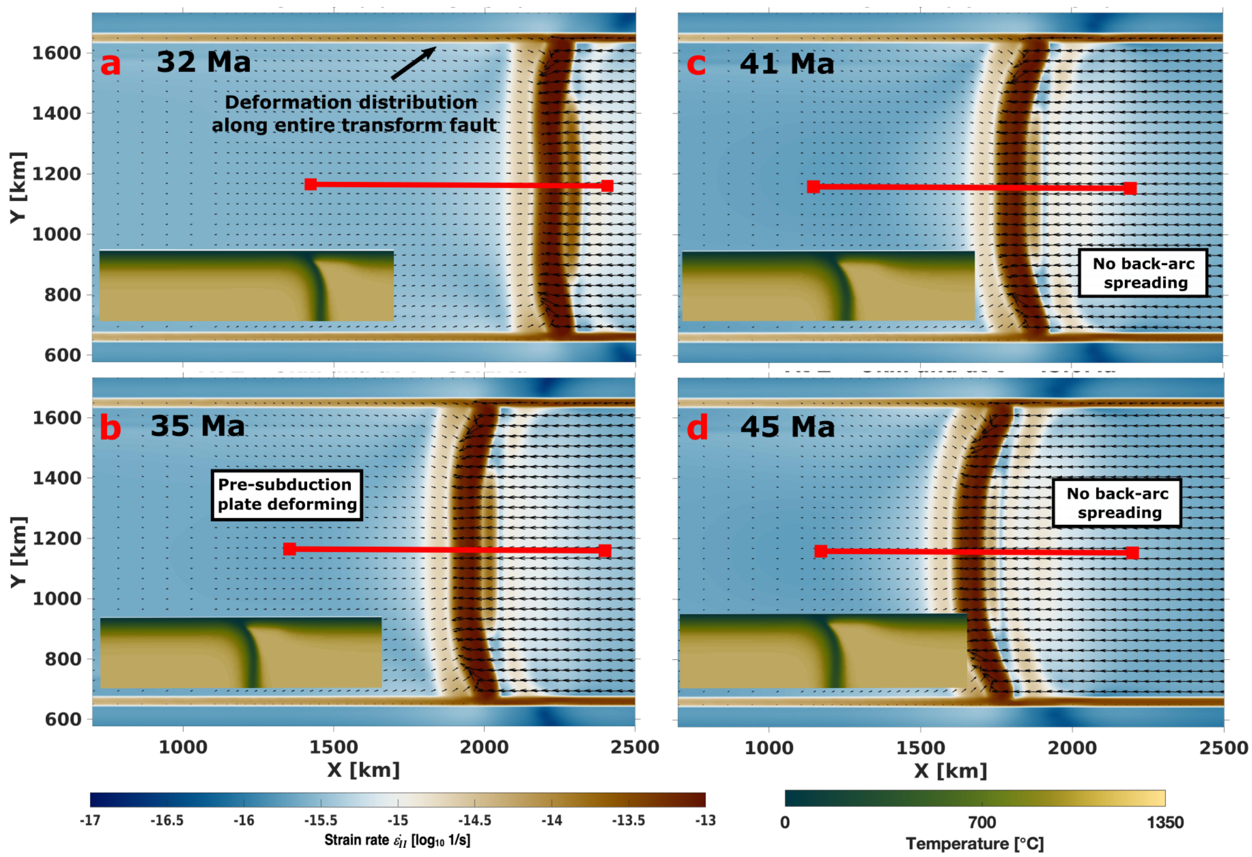

Supplementary Figure 6*. *Weak transform faults. *Evolution of a model with transform faults prescribed along the entire model domain. The model onset time is defined as t=0, because no back-arc basin opens at all. Without large tear resistance to be overcome, the subducting slab retreats continuously without rupturing the overriding plate and back-arc spreading jumps. A main difference with models that form transform faults self-consistently is that deformation is distributed along the entire plate margin, and not limited to the STEP faults and bounding transform faults.*

SUPPLEMENTARY DISCUSSION

The relationship between transform fault lengths, overriding plate widths, and the timing of back-arc spreading centre jumps.

The maximum distance from the back-arc spreading centre to the subduction zone and the period between back-arc ridge jumps are both linearly proportional to the width *W* of the overriding plate, for both observed and natural systems, and is marked by a linear regression

line (Figure 4). To explain this, we present the following first-order energy balance between frictional sliding at the transform faults and the breaking of the overriding plate to form a new back-arc spreading ridge.

The energy E_{OP} required to rift the overriding back-arc plate after cooling for a time t is proportional to its thickness T_{OP} and to its width W. To first-order, the thickness can be approximated as a halfspace cooling thickness, so $T_{OP} = A_1\sqrt{t}$, with A_1 a constant. So:

$$E_{OP} = A_2 W A_1 \sqrt{t} \qquad \text{(S1)}$$

with A_2 another constant.

The energy E_{TF} to keep the transform faults active depends on the length of the transform faults L and their thickness, or depth extent, which varies with distance to the back-arc spreading centre. To be more precise, E_{TF} is proportional to the transform fault surface area, which can be calculated as

$$E_{TF} = A_3 \int_{L\prime=0}^{L\prime=L} T_{OP} dL' = A_3 \int_{t\prime=0}^{t\prime=t} A_1\sqrt{t}\, dt' = \frac{2}{3} A_3 A_1 t^{\frac{3}{2}} \qquad \text{(S2)}$$

with A_3 yet another constant.

The ridge jump occurs when $E_{TF} = E_{OP}$:

$$A_3 A_1 t^{\frac{3}{2}} = A_2 W A_1 \sqrt{t} \qquad \text{(S3)}$$

or

$$t = \frac{A_2 W}{A_3} \qquad \text{(S4)}$$

In other words, the timing of the ridge jump t is expected to be linear proportional to the width of the back-arc plate W. Assuming a constant spreading rate, this also means that the back-arc plate length and width are proportional.

This analysis is simplified, as it makes the following assumptions: a) the friction across the transform fault is assumed to be uniform and not dependent on slip rate, depth, or temperature; 2) the tectonic stress inside the back-arc plate is assumed to be uniform; and 3) mantle drag at the base of the overriding plate may affect where the new back-arc basin will form, but does not significantly affect this energy balance.

SUPPLEMENTARY METHODS

3D numerical modelling follows the subduction setup in Magni et al. (2017) and Magni et al. (2014). We use a Cartesian version of Citcom[3,4], a finite elements code for mantle convection, which solves the conservation equations for mass, energy, momentum and composition:

$$\nabla \cdot \boldsymbol{u} = 0$$

$$\frac{\partial T}{\partial t} + \boldsymbol{u} \cdot \vec{\nabla} T = \vec{\nabla}^2 T$$

$$-\vec{\nabla} p + \vec{\nabla} \cdot \eta \left[\left(\vec{\nabla} u \right) + \left(\vec{\nabla} u \right)^T \right] + \left(Ra_T T - Ra_{C,i} C_i \right) \boldsymbol{e}_z = 0$$

$$\frac{\partial C_i}{\partial t} + \boldsymbol{u} \cdot \vec{\nabla} C_i = 0$$

See Table 1 for all parameters. The thermal Rayleigh number, Ra_T, and compositional Rayleigh number for a given composition i, $Ra_{C,i}$ are defined as

$$Ra_T = \frac{\alpha \rho g \Delta T\, h^3}{\kappa \eta}$$

$$Ra_{C,i} = \frac{g \Delta \rho_i\, h^3}{\kappa \eta}$$

While the flow field and diffusive term of the heat equation are solved on the finite element grid, the advective part of the temperature and composition equations are solved using approx. 2.000.000 numerical particles[5].

Model Setup

The size of the model domain is dependent on each model. All models are 660 km deep and 4620 km long. The width varies from 1720km (a 400km wide oceanic slab between two 660 km wide neighbouring continents) to 2820 km (a 1500km wide oceanic slab with two 660km wide neighbouring continents). The subduction process is attained by modelling two lithospheric plates converging towards each other.

Instantaneous onset of subduction is ensured by implementing a pre-existing subducting slab within the mantle (Figure 2a) and predefined lateral weak zones (with a viscosity of 10^{20}Pa s) decoupling subducting/overriding plates from neighbouring plates. The initially embedded lateral weak transform faults terminate 660 km from the initial trench position to allow self-consistent tear propagation beyond this point (Figure 2a and Supplementary Figure 1).

The overriding plate and neighbouring plates are assumed to be entirely of continental origin. They consist of an initially 40 km thick buoyant continental crust (Table 1) and the continental lithosphere is determined by a linear thermal gradient from 0 ºC at the surface to the mantle potential temperature at 120 km. The thickness of the oceanic lithosphere is determined by a half-space cooling model with an age of 80 Ma. Decoupling of subducting

and overriding plates is implemented by a thin weak zone with a fixed mantle viscosity of 10^{20} Pa s. The weak zone is free to move laterally to model trench rollback and advance.

Subduction is purely driven by internal buoyancy forces, i.e. no external forcing is applied at model boundaries. We apply free slip boundary conditions to the top and all four side boundaries and no-slip to the bottom boundary. To ensure high rollback rates, the subducting plate is fixed to the model boundary by a thermal boundary condition identical to the initial oceanic depth profile[6,7]. The boundary conditions at the neighbouring continental plates is a linear thermal gradient identical to the initial continental thermal field (i.e. to 120 km depth) and mantle temperatures below. The overriding plate of continental origin is free to move between a mid-ocean ridge (imposed by a hot thermal boundary) and the trench on the right. The top boundary is set to T=0°C and the side boundaries are insulating.

The total resolution is 436x56 elements in the xz-plane and 129-209 elements in y-direction, depending on the size of the model box. Mesh refinement is applied within the oceanic domain and along the lateral plate boundaries. The typical resolution ranges from 7x7x8 above 200km to 20x20x13km in areas with lower viscosity contrasts.

Rheology

In these models we use a simple visco-plastic rheology which includes diffusion creep and dislocation creep, as well as a depth-dependent pseudo-plasticity. A maximum viscosity η_{max} (set to 10^{23} Pa s) is applied. Diffusion and dislocation creep follow the following flow law:

$$\eta_{diff,disl} = A^{\frac{1}{n}}\dot{\epsilon}^{\frac{1-n}{n}}\exp\left(\frac{E}{nRT}\right)$$

With n = 1 for diffusion creep (i.e. strain-rate independent viscosity) and n = 3.5 for

dislocation creep[8]. All parameters are explained in Table 1.

Pseudo-plasticity is used to mimic brittle deformation. We use a simple depth-dependent yielding with a friction coefficient of 0.1. The resulting effective yielding viscosity is implemented as:

$$\eta_y = \frac{\min(\sigma_0 + \mu p, \sigma_{max})}{\dot{\epsilon}}$$

in which μ is the friction coefficient, σ_0 and σ_{max} are the surface and maximum yield stress, respectively and $\dot{\epsilon}$ the strain rate. The final viscosity is then defined as:

$\eta_{eff} = \min(\eta_{diff}, \eta_{disl}, \eta_y, \eta_{max})$

Supplementary Table 1. Symbols, units and default model parameters

**Parameters**	**Symbols**	**Value and Unit**
Rheological pre-exponent	A	3.6 x 10^9 [Pa^{-n} s^{-1}]
Activation Energy	E	360 [kJ/mol]
Rheological power law exponent	n	1(diff. c.), 3.5(disl. c.) [-]
Lithostatic pressure	p_0	[Pa]
Gas constant	R	8.3 [J/K/mol]
Temperature	T	[°C]
Compositional function	C	[-]
Velocity	u	[m/s]
Vertical unit vector	$\vec{e}_z$	[-]
Absolute temperature	T_{abs}	[K]
Reference temperature	T_m	1350 [°C]
Thermal Rayleigh Number	Ra_T	4.4x10^6 [-]
Compositional Rayleigh Number	Ra_C	1.7x10^7[-]
Gravitational acceleration	g	9.8 [m/s^2]
Thermal expansivity	α	3.5×10^{-5} [K^{-1}]
Thermal diffusivity	κ	10^{-6} [m^2/s]
Compositional density contrast	$\Delta\rho_c$	600 [kg/m^3]
Strain rate	$\dot{\epsilon}$	[s^{-1}]
Effective viscosity	η_{eff}	[Pa s]
Yielding viscosity	η_y	[Pa s]
Temperature drop over model	ΔT	1350 [K]
Friction coefficient	μ	0.1 [-]

Reference density	ρ	3300 [kg/m^3]
Yield stress	τ_y	[MPa]
Surface yield stress	σ_0	40 [MPa]
Maximum yield stress	σ_{max}	400 [MPa]
**Model Geometry**		
Domain depth	h	660 [km]
Domain length	l	4620 [km]
Domain width	w	1720-2820 [km]
Overriding Plate thickness	*Hop*	120 [km]
Mesh resolution		from 7x7x8 to 20x20x8[km^3]
Oceanic Slab Age	-	80 [Ma]
Continental crust thickness	*Hc*	40 [km]
Weak zone viscosity	η_{weak}	10^{20} [Pa s]

SUPPLEMENTARY TABLES

We here present the sources for the data plotted in Figure 4. Table 1 provides an overview of the literature where we obtained the spreading duration of every back-arc spreading centre. To measure the distance between trench and back-arc basin, we used reconstructions of the specific regions and measured the equivalent distance with Google Earth. An example is given in Figure 1.

**Spreading/rift centre jumps**	**Trench length [km]**	**Spreading/Rifting duration [Myr]**	**Distance trench-back arc spreading centre [km]**	**Source/*Comments***
West Scotia Ridge (to East Scotia Ridge)	750±20	13±7	840±130	Eagles & Jokat, (2014); Maldonado et al., (2014) *The large error in the spreading results from the segmentation of the back-arc spreading centre. The chosen spreading duration value results from the time difference between the West Scotia ridge forming (at 30 Ma) and the initial spreading of the East Scotia Ridge at 17 Ma (Eagles & Jokat (2014)). From 17 to 6 Ma spreading occurs in both basins simultaneously, but at 17 Ma spreading at the West Scotia Ridge reduced dramatically and extension by slab rollback was dominantly compensated by fast spreading of the East Scotia Ridge. The presence of two spreading centres active at the same time is different from our models where there is a switch-off of spreading in the older centre when the new centre starts. However, we consider the formation of the East Scotia spreading centre at 17 Ma as the time the ridge jump happens, since it does correspond to a time in which the overriding plate breaks closer to trench, as it is the case in our models. The large error bars acknowledge the overlap of spreading between the two ridges (Eagles & Jokat (2014)).*
Venezuela Basin (to Grenada/Tobago Basin)	800±50	14±8	830±40	Allen et al., (2019) *The large error results from the unclear duration of spreading in the Venezuelan Basin. The lower limit of ~6 Myrs is based on the beginning of rotation of the Caribbean to South America (~65 Ma, Mueller et al., 2019), which can lead to formation of a back-arc spreading centre (Magni et al., 2014), to the extinction of the associate Venezuelan volcanic Arc (~59 Ma, Neill et al., 2011). The upper limit (22 Myrs) is given by the maximum length of spreading as given in Figure S8 in the supplementary material of Allen et al., (2019).*
Cornaglia rifting (to Vavilov Basin)	280±20	7±3	265±20	Gueydan et al., (2017); Guillaume et al., (2010), and references therein
Vavilov (to Marsili Basin)	250±30	3±1	310±30	Gueydan et al., (2017); Guillaume et al., (2010), and references therein
**Active back-arc spreading**				Data below from Wallace et al., (2009) and references therein *Values are averaged if several times are given*
Lau Basin	1500	5.5	350	
Manus Basin	600	3.5	325	

Okinawa Trough	1200	6±2	235	
Bransfield Trough	650	4	170	
**Ceased back-arc spreading**				
Japan Sea	1400	10±5	700	
Sulu Sea	550	4	220	
Liguro Basin	560	9±2	260	

Supplementary Table 2. Data used in Figure 4.

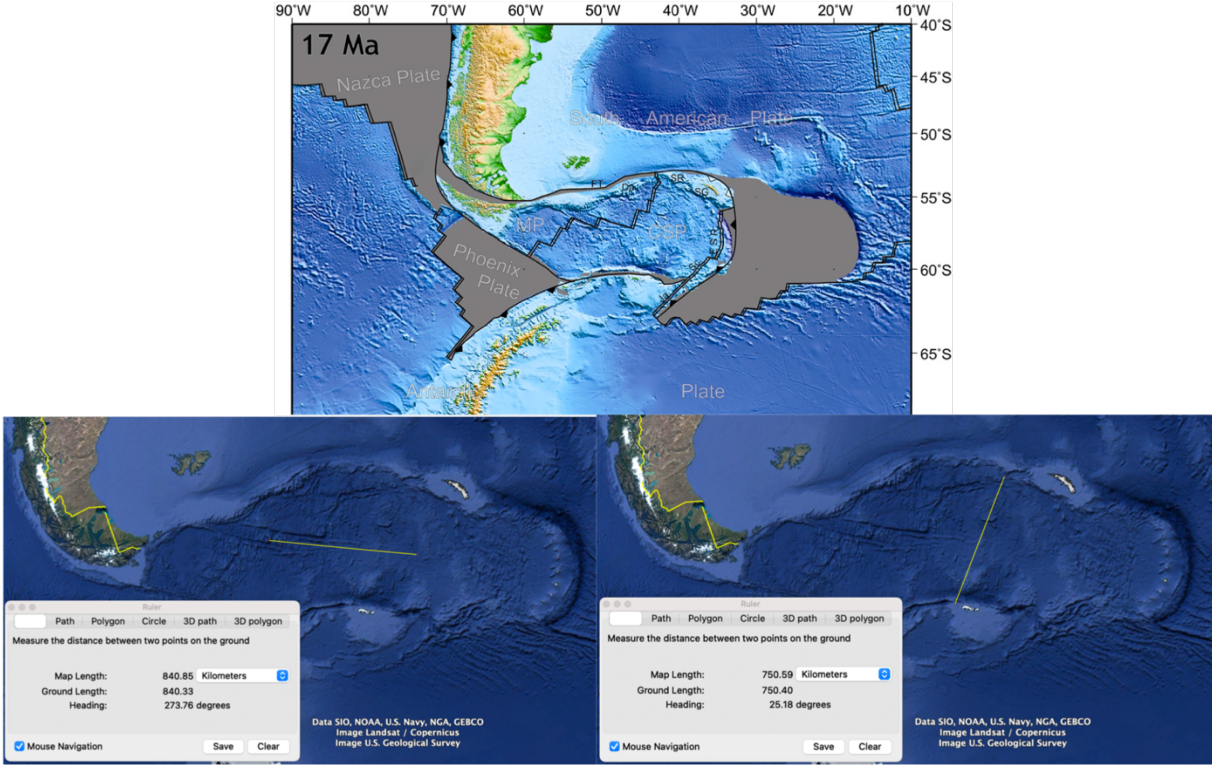

Supplementary Figure 7: *Example of measuring the distance between trench and back-arc spreading centre based on reconstructions (top, from Eagles & Jokat, (2014)) with Google Earth (bottom) in the Scotia Sea. All ceased back-arc spreading centres were measured with this method. The distance for the active basins were measured from the largest trench-perpendicular distance from trench to back-arc spreading centre.*

Bibliography

1. Magni, V., Faccenna, C., van Hunen, J. & Funiciello, F. How collision triggers backarc extension: Insight into Mediterranean style of extension from 3-D numerical models. *Geology* **42**, 511–514 (2014).
2. Magni, V., Allen, M. B., van Hunen, J. & Bouilhol, P. Continental underplating after slab break-off. *Earth and Planetary Science Letters* **474**, 59–67 (2017).
3. Moresi, L., Zhong, S. & Gurnis, M. The accuracy of finite element solutions of Stokes's flow with strongly varying viscosity. *Physics of the Earth and Planetary Interiors* **97**, 83–94 (1996).
4. Zhong, S., Zuber, M. T., Moresi, L. & Gurnis, M. Role of temperature-dependent viscosity and surface plates in spherical shell models of mantle convection. *Journal of Geophysical Research: Solid Earth* **105**, 11063–11082 (2000).
5. van Hunen, J., van den BERG, A. P. & Vlaar, N. J. On the role of subducting oceanic plateaus in the development of shallow flat subduction. *Tectonophysics* **352**, 317–333 (2002).
6. Clark, S. R., Stegman, D. & Müller, R. D. Episodicity in back-arc tectonic regimes. *Physics of the Earth and Planetary Interiors* **171**, 265–279 (2008).
7. Stegman, D. R., Schellart, W. P. & Freeman, J. Competing influences of plate width

and far-field boundary conditions on trench migration and morphology of subducted slabs in the upper mantle. *Tectonophysics* **483**, 46–57 (2010).
8. Ranalli, G. *Rheology of the Earth*. (Springer Science & Business Media, 1995).
9. Eagles, G. & Jokat, W. Tectonic reconstructions for paleobathymetry in Drake Passage. *Tectonophysics* **611**, 28–50 (2014).
10. Maldonado, A. *et al.* A model of oceanic development by ridge jumping: opening of the Scotia Sea. *Global and Planetary Change* **123**, 152–173 (2014).
11. Allen, R. W. *et al.* The role of arc migration in the development of the Lesser Antilles: A new tectonic model for the Cenozoic evolution of the eastern Caribbean. *Geology* (2019).
12. Gueydan, F., Brun, J.-P., Phillippon, M. & Noury, M. Sequential extension as a record of Corsica Rotation during Apennines slab roll-back. *Tectonophysics* **710**, 149–161 (2017).
13. Guillaume, B., Funiciello, F., Faccenna, C., Martinod, J. & Olivetti, V. Spreading pulses of the Tyrrhenian Sea during the narrowing of the Calabrian slab. *Geology* **38**, 819–822 (2010).
14. Wallace, L. M., Ellis, S. & Mann, P. Collisional model for rapid fore-arc block rotations, arc curvature, and episodic back-arc rifting in subduction settings. *Geochemistry, Geophysics, Geosystems* **10**, (2009).
